# Engineering Self‐Powered Electrochemical Sensors Using Analyzed Liquid Sample as the Sole Energy Source

**DOI:** 10.1002/advs.202203690

**Published:** 2022-08-18

**Authors:** Sunil Kumar Sailapu, Carlo Menon

**Affiliations:** ^1^ Biomedical and Mobile Health Technology (BMHT) lab Department of Health Sciences and Technology ETH Zürich Zürich 8008 Switzerland

**Keywords:** batteries, biofuel cells, electrochemical systems, enzymes, ions, ion‐selective electrodes, self‐powered sensors

## Abstract

Many healthcare and environmental monitoring devices use electrochemical techniques to detect and quantify analytes. With sensors progressively becoming smaller—particularly in point‐of‐care (POC) devices and wearable platforms—it creates the opportunity to operate them using less energy than their predecessors. In fact, they may require so little power that can be extracted from the analyzed fluids themselves, for example, blood or sweat in case of physiological sensors and sources like river water in the case of environmental monitoring. Self‐powered electrochemical sensors (SPES) can generate a response by utilizing the available chemical species in the analyzed liquid sample. Though SPESs generate relatively low power, capable devices can be engineered by combining suitable reactions, miniaturized cell designs, and effective sensing approaches for deciphering analyte information. This review details various such sensing and engineering approaches adopted in different categories of SPES systems that solely use the power available in liquid sample for their operation. Specifically, the categories discussed in this review cover enzyme‐based systems, battery‐based systems, and ion‐selective electrode‐based systems. The review details the benefits and drawbacks with these approaches, as well as prospects of and challenges to accomplishing them.

## Graphical abstract

Self‐powered electrochemical sensor (SPES) utilizes available chemical species in an analyzed liquid sample to generate response without using external power sources. This review discusses various sensing and engineering approaches with biofuel cell, battery, and ion‐selective electrode based SPESs toward formulating superior sensing platforms and freely behaving standalone devices that solely use liquid sample as power source.

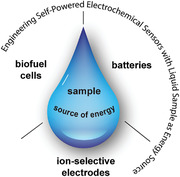

## 1 Introduction

Sensing devices are central to daily life, providing critical information about various physicochemical and biological parameters.^[^ ^1^ ^]^ This information enables for identifying a trend and adapting subsequent changes—indicating their crucial role in healthcare, environmental monitoring, agriculture, defense, and so on.^[^ ^2^, ^3^, ^4^, ^5^ ^]^ Amongst different techniques, electrochemical sensors that use the electrode as their transducing element^[^ ^6^ ^]^ have been widely employed toward quantification of various ions, bio‐, organic and inorganic molecules due to the availability of rapid, sensitive, selective and easy‐to‐use measuring tools.^[^ ^7^ ^]^ Most of these sensors require external electric fields to trigger favorable reactions at the electrode surface for enabling analyte detection.^[^ ^8^, ^9^ ^]^ However, in order to deploy these sensors in portable devices, point‐of‐care (POC) related platforms, implantable and wearable devices, their dependency on external power sources and high‐end instruments must be eliminated. Besides simplifying the sensor, this also requires considering compatible electronics, response indicators, signal transmitting modules and data management. Moreover, the power required to handle the entire device's operation remains a significant factor.^[^ ^10^ ^]^ On the one hand, the seemingly feasible solution offered by portable power sources like batteries come with a plethora of disadvantages. First, their rigid, heavy and over‐sized nature can be inconvenient to the user of wearable sensors.^[^ ^11^ ^]^ Second, the power a battery offers may also be surplus for disposable or single‐use POC devices.^[^ ^12^ ^]^ Third, dealing with complications from self‐discharge, limited shelf‐life, constant recharging or replacement would be a hassle especially in the case of implanted devices.^[^ ^13^, ^14^ ^]^ Fourth, the cost associated with these portable power sources further imposes constraints on its use, especially in countries with limited access to resources.^[^ ^15^ ^]^ Fifth, their uncontrolled disposal accumulates e‐waste, which is an environmental concern.^[^ ^16^, ^17^ ^]^ On the other hand, the alternative also comes with some disadvantages: implementing wireless excitation sources to trigger sensing reactions in electrochemical systems may result in large proximal sources, unwanted disturbances, power dissipation, leakage currents and also restricts independent device operation.^[^ ^7^, ^18^ ^]^

A promising alternative approach to handle this demand for power is to design a self‐powered electrochemical sensor (SPES) that utilizes the available chemical species in the operating body fluids such as blood, tear, saliva, sweat and interstitial fluid, or in environmental samples like river water for scavenging energy and generating a signal relevant to the information of analyte. A galvanic cell configuration can be used to formulate such an SPES by combining an indicator electrode with a second electrode to induce spontaneous thermodynamically favorable reactions.^[^ ^7^, ^8^ ^]^ Katz et al. introduced this approach in the scientific literature by demonstrating a “self‐powered biosensor (SPB)” for quantifying glucose using a biofuel cell with glucose oxidase (GOx) modified anode for promoting the oxidation of glucose and releasing electrons. A cytochrome c/cytochrome oxidase (Cyt c/COx) functionalized cathode in this biofuel cell helped to achieve the reduction of oxygen.^[^ ^19^ ^]^ As a consequence of these spontaneous reactions on exposure to glucose, the electrochemical cell generated a concentration‐dependent open‐circuit voltage (OCV) across the electrodes. Though the resultant power with these types of sensors is relatively low (typically in the order of microwatts), it still proved sufficient to trigger a sensing event and generate an electrical response without involving any external energy sources. These kinds of biofuel cell based SPBs use species of biological origin (e.g., enzymes, microorganisms, organelles) as catalysts and can function in profuse electrolytes making it feasible to apply them in complex physiological environments. Besides biocatalyst‐based fuel cells, battery‐based systems can also function as self‐powered sensors. For instance, the analyzed liquid sample can assume the role of an electrolyte in a traditional battery.^[^ ^13^ ^]^ This liquid sample‐based battery can generate an electrical response via spontaneous redox reactions using the electrolyte species. Moreover, widely employed ion‐selective electrode (ISE) systems that can quantify prominent ions (e.g., H^+^, Na^+^, K^+^) of biological and environmental significance^[^ ^20^ ^]^ can also be exploited toward developing self‐powered sensing systems, for example, by deriving a current while subjecting a potentiometric sensor to a non‐zero current operation.^[^ ^21^, ^22^ ^]^ All the above three electrochemical systems offer the advantage of a simple configuration with two electrodes, making miniaturization easy.^[^ ^23^, ^24^, ^25^ ^]^ Their ability to operate with bio‐related fluids opens possibilities for developing autonomous sensing devices, power sources for wearable and implantable devices.^[^ ^26^, ^27^ ^]^ Moreover, their tailored integration could result in inexpensive screening and disposable platforms.^[^ ^28^, ^29^, ^30^ ^]^

So far, reviews in the past briefed self‐powered electrochemical systems that derived energy through redox reactions using available chemical species in liquid sample. The majority of them covered biofuel cell configurations with focus on energy harvesting (e.g., for powering implantables, wearables) and self‐powered sensing of various analytes.^[^ ^8^, ^11^, ^26^, ^27^, ^31^, ^32^, ^33^, ^34^, ^35^, ^36^, ^37^, ^38^, ^39^ ^]^ Particularly, while reporting developments related to sensing, several of these reviews discussed different biofuel cell‐based systems (enzymatic, microbial, organelle, photocatalytic), involved electrode materials, immobilization techniques, energy and power densities, detection strategies based on molecule interactions (e.g., substrate effect, inhibition effect, enzyme effect, blocking effect), combined operation with electronic modules and electrochromic materials. However, a crucial element in advancement of SPESs is their evolution toward a completely free‐standing standalone device, where engineering approaches alongside sensing mechanisms remain critical. Moreover, while categorizing SPESs based on these strategies, it becomes essential to include other electrochemical sensing systems that target a similar objective, such as those based on liquid‐activated batteries and ion‐selective electrodes, particularly with exciting development in recent times. Alternative self‐powered systems such as those based on triboelectric nanogenerator (TENG) technique exploiting the liquid–solid contact electrification principle have also been explored toward energy harvesting and sensing purposes.^[^ ^40^, ^41^, ^42^ ^]^ These systems harvest mechanical energy from a liquid, via combination of electrification and electrostatic screening effect, to establish an electron transfer at the interface when a moving liquid typically contacts and separates from a material of different triboelectric polarity.^[^ ^43^, ^44^ ^]^ As these systems convert mechanical energy to electricity with an operating principle distinct from earlier described electrochemical systems (with biofuel cells, batteries, and ion‐selective electrodes) involving chemical species, we did not include them in the current review.

**Figure 1:**
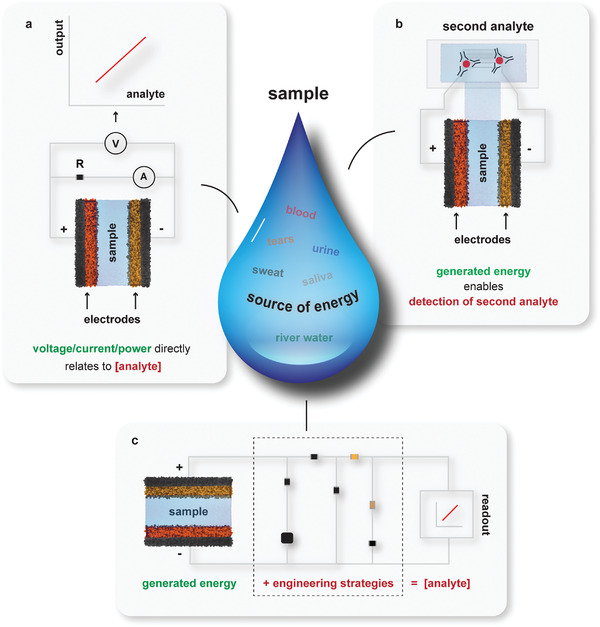
Self‐powered electrochemical systems utilizing analyzed liquid sample as energy source. a) Sensors that provide voltage/current/power directly relating to the analyte concentration. b) Sensors that generate energy using the first analyte to power another sensing platform designed to detect a second analyte. c) Realizing advanced sensor functionality or standalone device implementation by combining sensing and engineering strategies.

This review primarily focuses on discussing various sensing and engineering approaches adopted with SPESs operated using liquid samples by classifying different research works into three categories: 1) relating the voltage, current, or power (under a resistive load) generated with SPES directly to inform on the analyte (**Figure** **1**a);^[^ ^45^, ^46^, ^47^ ^]^ 2) enabling measurement of a second analyte in the same sample by supporting other sensing platforms with power generated from an SPES (Figure 1b);^[^ ^48^, ^49^ ^]^ and 3) combining different sensing and engineering approaches to derive superior sensing platforms (with on‐board memory, visual indicators, or wireless transmission) and self‐sufficient standalone devices (with on‐board digital response indicators) by incorporating an SPES (Figure 1c).^[^ ^12^, ^22^, ^29^, ^50^, ^51^, ^52^, ^53^ ^]^ The review introduces these strategies by citing relevant works with biofuel‐cell (enzymatic) based systems, battery‐based systems and ion‐selective electrode‐based systems. Besides discussing different sensing and engineering approaches, the review also indicates the advantages and disadvantages of these approaches, as well as potential opportunities and challenges.

## 2 Working Principle of Electrochemical Systems for Self‐Powered Sensing

Electrochemical systems dealing with the detection of metabolites often use biofuel cells.^[^ ^33^ ^]^ Biofuel cells are a sustainable technology that can function without expensive metal catalysts.^[^ ^54^ ^]^ They commonly use oxidoreductases, enzymes, organelles, or microorganisms as catalysts to aid the generation of electrical energy. Depending on the involved type of catalyst, the biofuel cells are classified as microbial fuel cells (micro‐organisms as catalyst),^[^ ^55^ ^]^ enzymatic biofuel cells (enzymes as catalyst),^[^ ^14^ ^]^ organelle biofuel cells (organelles as catalyst),^[^ ^56^ ^]^ or photocatalytic fuel cells (semiconductor photocatalyst).^[^ ^57^ ^]^ In this review, we focus on enzymatic biofuel cells due to the vast interest and their applicability, particularly in the field of biomedical engineering, arising from their ability to generate higher power densities and response solely using liquid samples based on specific catalytic effects.^[^ ^31^, ^35^, ^58^ ^]^ In addition, biological enzymes are highly specific, selective, sustainable, renewable, and possess excellent catalytic activity.^[^ ^27^, ^31^ ^]^ In an enzymatic fuel cell, these enzymes are used in isolated form without the cell membrane. Remarkably, though operated outside their native environment, these enzymes can still catalyze the oxidation of fuels such as glucose,^[^ ^19^, ^45^, ^47^ ^]^ lactic acid^[^ ^59^ ^]^ and ethanol,^[^ ^60^ ^]^ and the reduction of components like oxygen,^[^ ^23^ ^]^ meeting the needs for constructing a fuel‐cell. They further provide the opportunity to design miniaturized fuel cells forgoing any separator between electrodes due to the specificity of the involved reactions.^[^ ^26^ ^]^ Additionally, their biocompatible and non‐toxic nature suits the requirements of wearable and implantable devices.^[^ ^61^ ^]^

**Figure 2:**
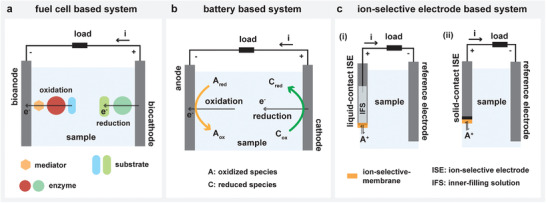
Electrochemical systems for self‐powered sensing. a) Fuel cell‐based system, b) battery‐based system, and c) i) liquid contact ion‐selective electrode‐based system and ii) solid‐contact ion‐selective electrode‐based system.

In a typical enzyme‐based biofuel cell, the fuel (e.g., glucose) gets oxidized at the anode with the help of enzymes (e.g., glucose oxidase) and releases electrons (**Figure** **2**a). The cathode promotes reduction of a suitable oxidant (e.g., oxygen) by collecting these electrons through an external circuit. Based on their mode of operation, biofuel cells are generally classified as direct electron transfer (DET) or mediated electron transfer (MET). To enable efficient electron transfer between electrode and enzyme redox center, the electron transfer rate must be as efficient as the biocatalytic reaction rate.^[^ ^11^ ^]^ To achieve this, biofuel cells based on MET use a small molecule or polymer based redox mediator. Employing suitable mediators in cell design would help in achieving higher current (and power) densities when combined with conductive materials.^[^ ^36^ ^]^ While in DET, this electron‐transfer happens directly between the electrode and catalyst. Under open‐circuit conditions, the voltage across the electrodes of biofuel cell can be predicted using the standard reduction potentials of the enzymes. However, in MET, these values will typically be lower due to the reduction potential differences between involved mediators and enzyme cofactors.^[^ ^62^ ^]^

Liquid‐activated batteries are another type of electrochemical system that can be adapted to function as a self‐powered sensor (Figure 2b). Similar to biofuel cells, these batteries feature an anode and cathode responsible for carrying out oxidation and reduction reactions respectively.^[^ ^28^ ^]^ However, unlike traditional batteries that host the electrolyte in a fused cell, these batteries only activate or operate upon introducing liquid samples such as water‐based fluids, beverages, or biological fluids. Thereby, the liquid sample acts as an electrolyte for this primary type of battery that invariably cease to function upon exhaustion of the electrodes.^[^ ^34^ ^]^ The voltage generated by the battery relies on the thermodynamics of the reactions occurring at the anode and cathode, respectively. On the other hand, several factors like electrolyte resistance, electrode resistance, double‐layer capacitance, and charge‐transport mechanism can influence the overall current provided by these batteries.^[^ ^46^ ^]^ Compared to most of the biofuel cells, liquid‐based batteries can achieve higher voltages, currents, and power (in the range of milliwatts per centimeter square). Both biofuel and batteries can generate power that is proportional to the concentration of the fuel and thereby can provide different ways of realizing self‐powered sensors.

ISEs are primarily used in potentiometric sensing, where the mode of measurement involves reading its potential against a standard reference electrode (whose potential is constant) under a nearly zero current condition.^[^ ^63^ ^]^ Typically, ISEs feature a perm‐selective membrane containing an ionophore that possess a high degree of selectivity to only one specific ion depending on the membrane composition (Figure 2c).^[^ ^64^ ^]^ In liquid‐contact ISEs, the membrane separates an inner‐filling (reference) solution from the sample. This typical construction of ISEs result in developing phase‐boundary potentials while the system seeks equilibrium. By incorporating an internal reference electrode in the inner‐filling solution, the potential developed across the membrane can be measured against a standard reference electrode. This induced voltage relates to the activity of detected ion according to the Nernst equation.^[^ ^65^, ^66^ ^]^ A solid‐contact, such as a conductive polymer replaces the inner‐filling solution in case of solid‐contact ISEs.^[^ ^67^, ^68^, ^69^ ^]^ As a potentiometric sensor, ISEs are usually operated at zero‐current condition. However, interesting self‐powered approaches can be developed by making the system deviate from this condition to produce a current (e.g., using a resistor or capacitor as electrical load) that can further aid in deriving different kinds of sensor response.^[^ ^70^ ^]^

## 3 Generated Voltage/Current/Power Reveals Analyte Information

**Figure 3:**
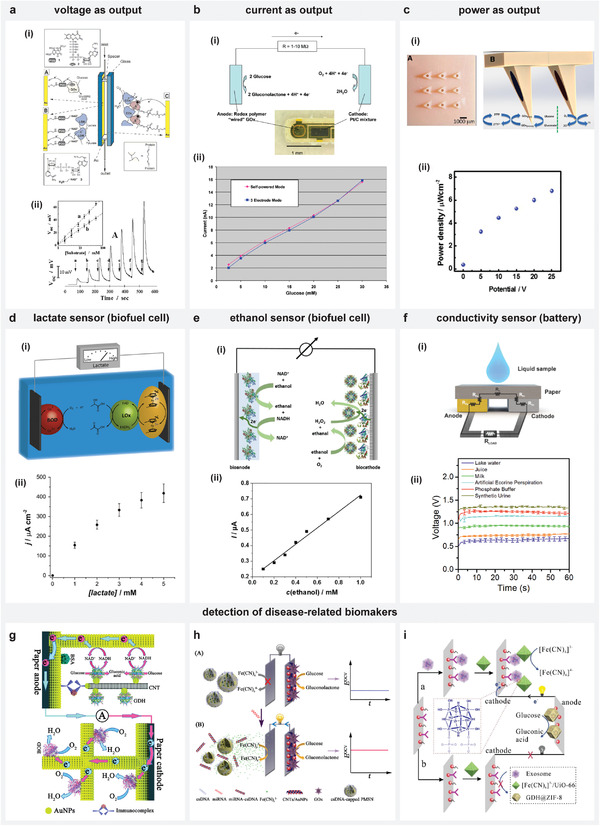
Generated OCV/current/power with SPES reveals information about analyte. a–c) Self‐powered glucose sensors with OCV, current, and power as output response. d,e) Biofuel cell‐based self‐powered sensors toward detection of biomarkers—lactate, ethanol respectively. f) Battery‐based self‐powered sensor with output voltage corresponding to conductivity of liquid sample. g–i) Self‐powered sensors toward detection of disease‐related markers—antigens, microRNA, and exosomes respectively. a) Reproduced with permission.^[^ ^19^ ^]^ Copyright 2001, American Chemical Society. b) Reproduced with permission.^[^ ^45^ ^]^ Copyright 2012, American Chemical Society. c) Reproduced with permission.^[^ ^47^ ^]^ Copyright 2014, Elsevier B.V. d) Reproduced with permission.^[^ ^59^ ^]^ Copyright 2015, Elsevier B.V. e) Reproduced with permission.^[^ ^60^ ^]^ Copyright 2017, Wiley‐VCH. f) Reproduced with permission.^[^ ^46^ ^]^ Copyright 2020, American Chemical Society. g) Reproduced with permission.^[^ ^75^ ^]^ Copyright 2014, The Royal Society of Chemistry. h) Reproduced with permission.^[^ ^77^ ^]^ Copyright 2018, The American Chemical Society. i) Reproduced with permission.^[^ ^78^ ^]^ Copyright 2020, Elsevier B.V.

The analyte can directly or indirectly contribute toward establishing thermodynamically favorable reactions to influence the voltage and/or current in SPES. As detailed earlier in the introduction of this review, the initial version of self‐powered sensor incorporating a biofuel cell used the analyte directly as a fuel. The oxidation of the available glucose in the sample propelled the sensor to generate a glucose‐concentration dependent OCV across the electrodes (**Figure** **3**a).^[^ ^19^ ^]^ However, utilization of this generated power further endows a substantial value to self‐powered sensors. By connecting an external load, a current flow can be established in the closed circuit forming the basis for a widely employed self‐powered amperometric mode of sensing. Liu et al. developed a glucose self‐powered sensor with the resistor as electrical load to the biofuel cell. The resultant output current through the resistor varied linearly with glucose concentration (Figure 3b).^[^ ^45^ ^]^ The biofuel cell accomplished this glucose‐concentration controlled current flow through the reduction of O_2_ at Pt/C cathode and the oxidation of glucose at GOx‐based anode. A similar mechanism‐based, miniaturized, and low‐cost self‐powered glucose sensor has also been achieved on a paper‐based platform.^[^ ^71^ ^]^ Further, electric power being related to the current and voltage, the analyte concentrations have also been revealed based on the power delivered by the SPES. Valdes‐Ramirez et al. demonstrated a hollow microneedle‐based glucose self‐powered sensor by incorporating a GOx‐based anode and a Pt black‐modified cathode to obtain analyte‐concentration dependent power densities (Figure 3c).^[^ ^47^ ^]^ Their sensor delivered power densities varying between 3 and 7 µW cm^−2^ for concentrations relevant to diabetes monitoring (5–25 mm).

The detection of important metabolites can be realized by relating the analyte concentrations with current/power densities obtained with an SPES. For instance, lactate detection has been achieved using an amperometric sensor with a lactate oxidase (LOx)‐immobilized anode (Figure 3d). The anode promoted the oxidation of lactate fuel to generate a lactate‐concentration dependent current when coupled with a bilirubin oxidase (BOD)‐based biocathode that accepted the produced electrons through the reduction of molecular oxygen.^[^ ^59^ ^]^ The sensor detected lactate concentration between 0 and 5 mm with a sensitivity of 45 ± 6 µA cm^−2^ mm ^−1^ and delivered a power density of ≈122 ± 5 µW cm^−2^. Neurotransmitters such as acetylcholine (ACh) are prominent biomarkers in brain disorders such as Alzheimer's.^[^ ^72^ ^]^ The self‐powered detection of ACh was made possible by studying the current profile of the biofuel cell under the influence of an external load resistor of 80 kΩ. The fuel cell constructed with acetylcholinesterase (AChE) enzyme‐based anode (to promote the oxidation of ACh) and a Pt‐based cathode (to promote O_2_ reduction) delivered a maximum power of 4 nW for a current density of 9 µA cm^−2^ with 10 mm ACh. The current through the resistor varied linearly with ACh concentration and delivered a sensitivity of 0.1 µA mm ^−1^ cm^−2^ with a lower detection limit of 10 µm.^[^ ^73^ ^]^ Ruff et al. employed the self‐powered amperometric based sensing approach to detect ethanol, a prominent component in liquors, drugs and biological samples by short‐circuiting the biofuel cell (Figure 3e). Their sensor used two ethanol‐converting electrodes, that is, a bioanode with nicotinamide adenine dinucleotide hydrate (NAD^+^)‐dependent alcohol dehydrogenase (ADH), a biocathode with alcohol oxidase (AOx) and horseradish peroxidase (HRP). The short‐circuited current in the presence of ethanol varied linearly with concentration in the range of 0.1 to 1 mm.^[^ ^60^ ^]^ With a similar approach, Sekretaryova et al. developed a self‐powered sensor for detecting cholesterol, an important biomarker for atherosclerosis and lipid‐related disorders.^[^ ^74^ ^]^ Interestingly, they achieved this by incorporating the same enzyme, that is, cholesterol oxidase (ChOx) on both the anode and the cathode and obtained a cell that delivered a maximum power density of 11.4 µW cm^−2^. Additionally, the cathode contained Prussian blue (PB) that helped in electrocatalytic reduction of H_2_O_2_ produced during the conversion of cholesterol by ChOx. The short‐circuited circuit during sensor operation related linearly to cholesterol concentration between 0.15 and 4.1 mm with a sensitivity of 26.0 ± 0.5 mA m ^−1^ cm^−2^ and detection limit of 1.4 µm. Besides biofuel cells, a paper‐based battery has also been reported for measuring the conductivity of real liquid samples such as milk, juice, artificial eccrine perspiration, urine, lake water, and phosphate buffer. With laterally placed Mg and Ag/AgCl electrodes as anode and cathode, Ortega et al. developed a self‐powered conductivity sensor that used these analyzed liquid samples as an electrolyte. These electrodes, when connected externally through a suitable resistor, resulted in a stable voltage depending on the conductivity of the sample (Figure 3f).^[^ ^46^ ^]^

Affinity‐based biorecognition aids in detecting diseases‐related markers and can be accomplished by tagging them to suitable compounds that can influence the electrochemical reactions at the electrode surface. For instance, Wang et al. developed a paper‐based self‐powered immunosensor by integrating a sandwich‐immunoassay in a biofuel cell operation for detecting cancer‐related carcinoembryonic antigen (CEA) in serum (Figure 3g). The gold nanoparticle (AuNP)‐modified anode contained the capture antibodies (Ab1) to bind target CEA. The cathode was coated with bilirubin oxidase (BOD) to promote the oxygen reduction required for the biofuel cell to operate. On loading the glucose dehydrogenase (GDH)‐conjugated signal antibody (Ab2), an immunocomplex forms only if CEA was initially bound to Ab1. To quantify Ab2 (and CEA), glucose with an NAD^+^/NADH cofactor was used as the fuel for the biofuel cell. The GDH in the formed immunocomplex catalyses glucose oxidation while oxygen gets reduced at the BOD‐based cathode. This results in a current through an external circuit that depended on the concentration of captured CEA.^[^ ^75^ ^]^ The sensor response varied linearly with logarithmic concentration of CEA (0.001–1000 ng mL^−1^) with a limit of detection corresponding to 0.85 pg mL^−1^ and delivered a maximum power density of 130.7 µW cm^−2^ in presence of 100 ng mL^−1^. Further, based on the amount of loading of the enzyme substrate responsible for electrochemical reaction, Gai et al. developed a self‐powered sensing platform for detecting cancer. The biofuel cell‐based sensor consisted of a BOD‐bioconjugate‐aptamer modified cathode to catalyze oxygen reduction and a GDH‐based anode to catalyze glucose oxidation. In the presence of cancer cells, the aptamer released the BOD‐bioconjugate by undergoing a conformation change to establish a connection with cancer cells and capture them. As a result, the loading of BOD responsible for catalytic reduction of oxygen reduced resulting in a decrease of OCV (0.54 V) based on the number of cancer cells in the sample.^[^ ^76^ ^]^ Another important biomarker for diseases like cancer is microRNA (miRNA), as its expression levels provide critical information about the disease condition. Thereby, toward bio‐assaying of miRNA, a self‐powered ultrasensitive sensor has been achieved based on trapping and release of the electron acceptor in a biofuel cell (Figure 3h). The cathodic electron acceptor, which is [Fe(CN)_6_]^3−^ in the sensor was initially entrapped in positively charged mesoporous silica nanoparticles (PMSN) and capped with biogate DNAs which are complementary to the target miRNA. On detecting miRNA, the DNA forms a rigid DNA‐RNA hetero‐duplex structure separating it from positively charged mesoporous silica nanoparticles (PMSN). This resulted in a controlled release of [Fe(CN)_6_]^3−^ that eventually underwent reduction by accepting the electrons generated through the oxidation of glucose (catalyzed by the GOx‐based anode). The OCV generated in the process increased in relation to the detected miRNA, which in their case resulted in a limit of detection for miRNA‐21 assay down to 2.7 am.^[^ ^77^ ^]^ Recently, a self‐powered sensor has also been proposed for detecting exosome levels in biological fluids that likely associate with cancer or other diseases.^[^ ^78^ ^]^ This sensing system incorporated a metal‐organic framework (MOF) based design with GDH@zeolitic imidazolate framework‐8 (GDH@ZIF‐8) as an anode. A CD63 antibody at the cathode captured the exosomes, that further allowed Zr‐MOF loaded with K_3_[Fe(CN)_6_] molecules ([Fe(CN)_6_]^3−^/UiO‐66) to immobilize at the cathode surface through Zr—O—P bonds. This connectivity established through exosomes enabled the [Fe(CN)_6_]^3−^/UiO‐66 to accept electrons generated through the catalytic oxidation at the anode. The output voltage obtained with this sensing system increased with exosome concentration and achieved a detection limit of 300 particles per milliliters. Based on the sensing technique, the authors detected exosomes derived from cancer in complex biological media (Figure 3i).

In addition, biomarkers such as single nucleotide polymorphisms (SNPs), antibiotics, and many other bio‐ and environmentally relevant markers have been detected based on the dependence of voltage/current/power on analyte concentration.^[^ ^79^, ^80^, ^81^, ^82^, ^83^, ^84^ ^]^ With a simple design of the sensor incorporating only a couple of electrodes to operate with the analyzed liquid samples, SPES provides an electrical output that can help to decode the information about the analyte. While a crucial element in the sensor's simplification results from requiring no external power supply for triggering reactions, their implementation can be enhanced further by transforming them into a completely self‐sufficient and standalone device featuring easy readout indicators and without involving any complicated or cumbersome measuring equipment.

## 4 Generated Energy Enables Measurement of Second Analyte

Often, the analyzed sample is a composition of many ions, inorganic, and organic molecules. This presents the opportunity to conveniently utilize any of these available resources to extract the energy from spontaneous chemical reactions. So, even if the analyte of interest is not redox‐active to supply power for the detection platform, its sensing can be supported by generating power using other entities in the sample. Additionally, such a combination would enable fabricating robust self‐powered sensing platforms that can feature a variety of detection techniques entirely run with the power generated by the sample itself in an electrochemical cell.

**Figure 4:**
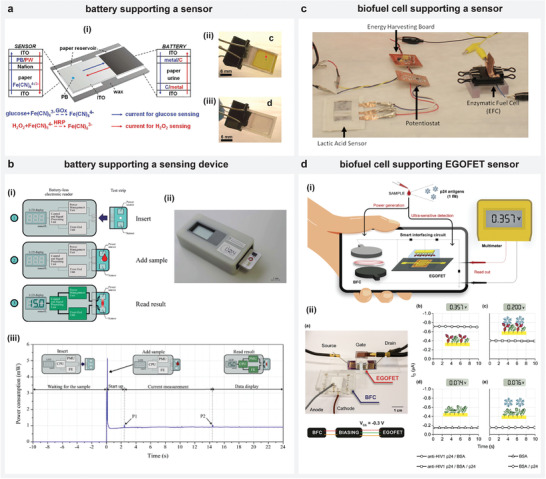
Generated energy enables measurement of second analyte. a) i) Scheme illustrating a urine‐sample activated Al/air battery driving another sensor measuring glucose in the sample, and a color‐based indicator, (ii) and (iii) PB color transition from blue to white giving qualitative information about glucose in an analyzed sample. b) i) Scheme illustrating “Plug‐and‐Power” concept, with a paper‐based battery power source and glucose sensor, (ii) and (iii) picture and working stages of the device. c) Picture showing a glucose bio‐fuel cell powered lactate sensing using sweat sample as energy source. d) i) Scheme representing the approach of glucose biofuel cell‐powered immunosensing with ultra‐sensitive EGOFET‐based sensor. ii) Picture of the sensing platform and its output response. a) Reproduced with permission.^[^ ^48^ ^]^ Copyright 2012, American Chemical Society. b) Reproduced with permission.^[^ ^85^ ^]^ Copyright 2018, Elsevier B.V. c) Reproduced with permission.^[^ ^86^ ^]^ Copyright 2016, The Electrochemical Society. d) Reproduced with permission.^[^ ^49^ ^]^ Copyright 2020, Elsevier B.V.

Liu et al. demonstrated a micro‐electrochemical sensing platform by incorporating an on‐board power source to drive an electrochemical sensor and a color‐based response indicator (**Figure** **4**a).^[^ ^48^ ^]^ The on‐board power source in the paper fluidics‐based sensing platform is an aluminum (Al)/air battery that activates upon introduction of the artificial urine sample. The power sources generated an average OCV of 0.94 V and onset short‐circuit current of 60 µA mm^−2^ that decreased gradually to 8 µA after 5 min. The detection principle is based on oxidizing glucose in the urine sample with preloaded glucose oxidase (GOx) in the paper reaction zone alongside reduction of Fe(CN)_6_^3−^ to Fe(CN)_6_^4−^. This reduced form of Fe(CN)_6_^4−^ oxidizes back to Fe(CN)_6_^3−^, triggering the transition of an electrochromic indicator, PB to Prussian white (PW) giving qualitative information about glucose. Thereby, the artificial urine sample acted here as both the electrolyte for the battery and the matrix for the analyte. In fact, the power generated using low volumes of body fluid samples can accomplish sensor measurements that often require a complex instrument like a potentiostat. Montes‐Cebrián et al. developed a POC self‐powered portable blood glucose meter featuring a glucose‐sensor, application‐specific electronic reader with a digital display indicator that is entirely run with a minimum serum volume of 12.5 µL (Figure 4b).^[^ ^85^ ^]^ Incorporating a Plug‐and‐Power concept, the device used a disposable test‐strip that included a paper‐based power source and a paper‐based glucose sensor. The power source only activated upon meeting physiological fluids such as serum or deionized water and adopts a proprietary battery model. For the purpose of glucose sensing, the power source produced an output voltage of 1.5 V and a minimum power of 10 mW for at least 20 min on using 12.5 µL of serum sample. The power source was connected to a power management circuit that further up‐scaled this voltage to 3 V required for driving other electronic modules in the device, both for sensing and for displaying the results on screen. The glucose sensor is a biofuel cell with a GDH‐based anode and a BOD‐based cathode. The device measured glucose concentration by performing a chronoamperometric measurement to obtain current by operating a potentiostat amplifier circuit using the on‐board power source. Overall, the device detected glucose concentrations between 5 and 30 mm, which is on par with commercial instruments. Thereby, the energy extracted from the fluid sample using battery chemistry allowed not only for the sensing of a different analyte incorporating biofuel cell and other electronic modules, but also to present the analyte concentration on a digital display.

Besides battery‐based systems, the power generated from biofuel cells also enabled the development of self‐powered sensing platforms. Garcia et al. used a glucose biofuel cell to generate sufficient power for operating an energy harvester and micro‐potentiostat for performing chronoamperometric detection of lactate in sweat.^[^ ^86^ ^]^ By employing a glucose‐oxidizing GOx‐based electrode alongside an oxygen‐reducing cathode, the glucose biofuel cell achieved over 80 mA of current at 0.4 V (Figure 4c). The energy harvester further converted this voltage to the 3 V required for operating a micro‐potentiostat. When operated by the micro‐potentiostat, the lactate sensor based on lactate dehydrogenase (LDH) achieved a linear response between concentration limits of 5 and 100 mm with a sensitivity of 0.2 µA mm ^−1^. Since sweat is made up of both glucose and lactate, this approach is promising where power derived from glucose can be used to support lactate sensing. In addition, the power generated from biofuel cell can drive ultra‐sensitive immunosensing platforms to enable early‐detection of infections such as HIV‐1. Sailapu et al. developed a self‐powered platform by coupling a paper‐based biofuel cell to power an electrolyte‐gated field‐effect transistor (EGOFET)‐based sensor through a smart interfacing circuit featuring simple electronic components (Figure 4d).^[^ ^49^ ^]^ The biofuel cell generated an OCV of ≈0.85 V with a sample volume of 3.5 µL, where the polarization curve revealed a maximum power of 3.36 ± 0.06 µW. Under proper biasing with suitable voltage, the three‐terminal based EGOFET provided a current response based on the interaction of an HIV‐1 p24 antigen contained in the sample against the functionalized antibody on the gate electrode. However, to enable this detection, the glucose‐based biofuel cell extracted the required energy from the analyzed sample to not only provide biasing for the EGOFET operation but also to deliver an easy voltage readout of sensor response with the help of the smart‐sensing circuit. By using this self‐powered platform, the authors detected the HIV‐1 p24 antigen in a femtometer range suitable for early‐stage diagnosis of HIV‐1 infection.

We have presented a few more works of platforms achieving analyte detection utilizing the power generated from a different resource in the same sample, such as in cascaded bipolar configurations, at various places in this review.

## 5 Engineering Strategies for Self‐Powered Sensing

Though the ability to obtain analyte information without involving any excitation sources such as a potentiostat is encouraging, the inadequate power from a single SPES poses challenges for their further implementation toward obtaining an advanced form of sensor or a self‐sufficient standalone device. The loading and redox potentials of the anode and cathode components (featuring cofactors, mediators), operating region, limited sample volumes, and the kinetics of the electrochemical process largely limit the sensor's OCV (often < 1 V) and associated currents (generally in the order of microamperes) to drive any auxiliary electronic components. As a result, the often‐observed low power densities (usually in the order of microwatts to a few milliwatts per centimeter square)^[^ ^87^, ^88^ ^]^ makes it difficult to operate a majority of commercial electronic devices and circuits—especially in miniaturized systems—solely by leveraging the energy derived from the analyzed liquid sample. Thereby, special strategies must be developed to render these sensors with advanced functions or to project them as a self‐sufficient standalone device with output indicators.

### 5.1 Harvesting Energy in a Capacitor

Besides the resistor, one of the simplest electrical loads that can be directly interfaced to the self‐powered sensor is the capacitor. Unlike a resistor that dissipates energy, the nature of capacitor allows for energy storage and to further manifest different ways of its practical use. A capacitor connected directly across the sensor terminals accumulates charge on its conductive plates (that are separated by a dielectric material) by establishing a transient current in the closed‐circuit. A voltage develops across the capacitor due to the accumulated charge that gradually approaches a steady‐state value, typically close to the OCV.^[^ ^21^ ^]^ The current almost ceases to flow in the circuit at steady‐state condition and the capacitor preserves the accumulated charge (stores energy) until discharged through an appropriate circuit path connecting to a load.

**Figure 5:**
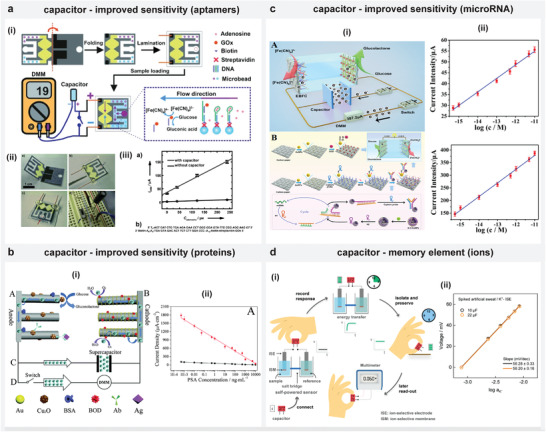
Harvesting energy in a capacitor. a) i) Scheme illustrating the operation of self‐powered adenosine sensor by initially harvesting energy in a capacitor to subsequently discharge it to read current through DMM, ii) picture of the sensor, iii) output current with and without a capacitor. b) i) POC self‐powered immunoassay for PSA detection with Ti‐based supercapacitor. ii) Response without using capacitor (left) and with capacitor (right). c) i) Scheme illustrating microRNA sensor interfaced to a capacitor, ii) output response without the capacitor (top) and with capacitor (bottom). d) i) Scheme illustrating ISE‐based self‐powered sensor to preserve response in capacitor for later readout, ii) response of the sensor in artificial sweat. a) Reproduced with permission.^[^ ^89^ ^]^ Copyright 2012, Wiley‐VCH. b) Reproduced with permission.^[^ ^90^ ^]^ Copyright 2015, The Royal Society of Chemistry. c) Reproduced with permission.^[^ ^62^ ^]^ Copyright 2022, American Chemical Society. d) Reproduced with permission.^[^ ^21^ ^]^ Copyright 2020, American Chemical Society.

An advantage of storing energy in a capacitor is the ability to discharge it by expending energy across various types of loads. For instance, when compared to the current extracted directly from SPES, an instantaneous current of greater magnitude can be generated by discharging a charged capacitor of similar voltage through a smaller value of resistive load. Liu et al. demonstrated that an instantaneous current measurement obtained by connecting a digital multimeter (DMM) across a charged capacitor provided a higher sensitivity in detection when compared to a direct current measurement without the capacitor.^[^ ^89^ ^]^ In brief, the paper‐based self‐powered adenosine sensor featured an area for sample inlet that were further split into two separate channels terminating in an hour glass‐shaped two‐compartment cell (**Figure** **5**a). One of these channels contained aptamer‐immobilized microbeads as a sensing probe while the other channel with only microbeads served as control. When the sample travelled through the channels, the aptamer bound to the target in one of these channels and released a GOx‐labelled DNA strand. This released GOx‐containing sample helped in catalyzing the oxidation of glucose on reaching the two‐compartment cell and converted [Fe(CN)_6_]^3−^ to [Fe(CN)_6_]^4−^ in one of the half‐cells. The induced voltage due to the concentration differences between [Fe(CN)_6_]^3−^ and [Fe(CN)_6_]^4−^ in the separated half‐cells was harvested in the form of energy by connecting a capacitor across the electrochemical cell. When this charged capacitor was connected to a less resistive DMM after 10 min with the help of a switch, an instantaneous current appeared with a magnitude depending on the adenosine concentration. By quantifying this current, they achieved a 17‐fold enhancement in sensitivity (0.48 mA mm^−1^) when compared to measurements without the capacitor (0.029 mA mm^−1^). Instead of a commercial electronic capacitor, Wang et al. incorporated an all‐solid‐state paper supercapacitor to achieve sensitive detection of DNA using a biofuel cell based on AuNPs‐modified anode and Pt‐modified cathode.^[^ ^81^ ^]^ Based on the hybridization status between capture and target DNA on the anode surface, the labeled Signal ssDNA containing bioenzymes GOx and HRP promoted oxidation and reduction reactions in the presence of glucose generating a current flow through the connected paper‐based supercapacitor. Typically, the paper‐based BFC delivered an OCV of 0.59 V and a maximum power density of 150 µW cm^−2^ with hybridization of 10 pm target DNA. The stored energy in the supercapacitor was released by connecting a DMM. The current obtained through this approach delivered a linear detection range of 10 fm to 100 nm with a detection limit of 6.3 fm for target DNA. Based on a similar approach, Gao et al. developed a POC self‐powered immunoassay toward detection of prostate‐specific‐antigen (PSA) by using a glucose‐air based biofuel cell with antibodies (Ab_1_) embedded Cu_2_O‐TiO_2_ nanotubes‐based anode, and a bilirubin oxidase (BOD) embedded Au‐TiO_2_ nanotubes‐based cathode (Figure 5b).^[^ ^90^ ^]^ The cell produced energy through oxidation of glucose with Cu_2_O‐TiO_2_ nanotubes and reduction of O_2_ to H_2_O with BOD‐Au‐TiO_2_ nanotubes. However, the interaction between the antibodies with the captured PSA in the sample hindered the diffusion of glucose to the anode and thereby modulated the current response across a solid‐state Ti supercapacitor. The release of the harvested energy in the capacitor upon connecting a DMM produced a ≈16.5‐fold increment in current response when compared to that obtained directly with biofuel cell. Recently, Wang et al. constructed an ultra‐sensitive platform for detecting microRNA‐21 by studying the influence of different parameters of a capacitor on the resultant current in the sensing system.^[^ ^62^ ^]^ Their biofuel cell contained a GOD/AuNPs/carbon paper (CP) based anode to generate electrons through catalytic oxidation of glucose (Figure 5c). The electroactive K_3_[Fe(CN)_6_], intended to collect these generated electrons, was inserted into mesoporous AuNP‐modified N‐doped carbon nanoshells (N‐C/AuNPs) and was tagged to an oligonucleotide H2. When target microRNA sequence was detected, H2 formed an assembly with another oligonucleotide H1 to eventually generate a “Y”‐shaped DNA complex with the capture probe on the cathode surface. This process of recycled amplification of target via hairpin assemblies of H1, H2 allowed immobilization of K_3_[Fe(CN)_6_] on the cathode. To transfer energy, a suitable capacitor was integrated to this biofuel cell by optimizing the charging time, voltage, and capacitance values. The derived instantaneous current from the charged capacitor showed an enhancement in detection sensitivity from 2.3 to 38.72 µA pm ^−1^ when compared to a biofuel cell's response without capacitor.

Another advantage of harvesting energy in a capacitor is the ability to use it as a memory element by preserving the response to enable a later readout. Sailapu et al. demonstrated this approach toward detecting the K^+^‐ion in simulated sweat samples using an electronic capacitor as a portable transduction component (Figure 5d).^[^ ^21^ ^]^ Their system consisted of a pair of identical K^+^‐ISEs placed in separate compartments, where one ISE (indicator electrode) is responsive to K^+^‐ion concentration in sweat sample. The other ISE (reference electrode) immersed in a solution of constant composition served as a reference. By connecting an electronic capacitor between these ISEs, the capacitor charged by virtue of a transient current generated due to the induced voltage between the electrodes arising from the ion concentration differences between the compartments. When this charged capacitor was physically isolated from the overall system, it maintained the stored energy that corresponded to the measured K^+^‐ion activity for hours. A measurement of this capacitor voltage using a simple handheld multimeter revealed the ion concentration in a physiologically relevant concentration range without requiring any high input impedance measuring instruments. Further, the ability to store the response for hours in a portable component like a capacitor enables a later readout at a convenient time without being in the proximity of the actual sensor itself.

Due to the low power available from SPES, the regular use of high‐end instrumentation to measure the resultant weak currents makes the system complicated for POC‐related applications. The above examples utilizing the capacitor to preserve the response and to generate higher magnitude currents allow for measurements with relatively simple, less expensive, and portable instrumentation. However, the maximum voltage that the capacitor can be charged to by this approach is limited to the OCV of the electrochemical cell. This is typical to the case of an electronic circuit interfacing a capacitor to a constant voltage source, where the maximum capacitor voltage achieved at a steady‐state condition is nearly the magnitude of the constant voltage source.^[^ ^91^ ^]^

### 5.2 Interconnecting Cells

A simple strategy to obtain higher voltages and greater currents with SPES is to interconnect a multiple number of individual cells. For instance, the voltage and current from a single biofuel cell, comprising a PQQ‐dependent GDH (PQQ‐GDH) and oxygen‐reducing laccase for the oxidation of glucose and the reduction of oxygen, has been improved by connecting individual cells either in series or parallel.^[^ ^92^ ^]^ While a single cell reported an OCV of 300–400 mV and short‐circuited current (*I*_sc_) of 30–100 µA, the series and parallel connection of three of these cells increased the OCV to 800 mV and *I*_SC_ to 300 µA, respectively. Paper‐based glucose/O_2_ enzymatic cells have been showed to achieve OCV of 2.65 V and maximum power of 350 mW at 1.55 V by connecting a series of five individual cells.^[^ ^93^ ^]^ The power and voltage thus generated proved sufficient to directly illuminate a light emitting diode (LED). The strategy of interconnecting cells offers the inherent advantage of packing cells in different configurations and sizes based on the required electric parameters of interest. While the overall power can be increased through this approach, it is also crucial to provide sensors that derive a proportional increment in their sensing response in such configurations. Similarly, a simple series connection of identical ISEs immersed in separate solutions can deliver a higher induced voltage with an enhanced sensitivity in response.^[^ ^94^ ^]^ The Nernstian response for potassium, calcium, nitrate, and carbonate ion‐selective electrodes has been showed to double and triple when a series connection was established between two and three electrodes, respectively, encouraging further SPES development. However, since the overall function of these interconnected cells rely on the efficient working of the individual units, parameters such as electrolytic or ionic short‐circuit currents, limited sample volumes and spatial distribution of the cell components may cause deviations in output signal with a magnitude different from the ideal theoretical value.^[^ ^95^, ^96^ ^]^

### 5.3 Bipolar Electrode Configurations

In a general sense, a bipolar electrode in an ionic medium is a conductor capable of developing a potential difference between its ends on applying a suitable voltage across it using a pair of separate electrodes in a non‐contact manner. Self‐powered operation of this bipolar element alone can be accomplished through spontaneous electrochemical reactions, say by simply short‐circuiting a cathodic element of suitably higher reduction potential than the anodic element and thereby avoiding the need for any driving electrodes or external polarization.

**Figure 6:**
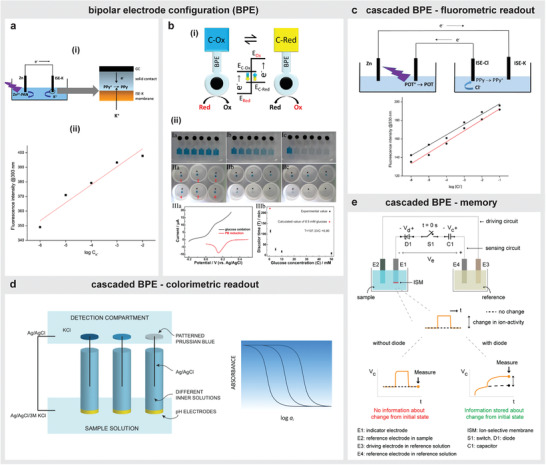
Bipolar and cascaded bipolar electrode systems. a) i) Scheme illustrating the working of self‐powered bipolar electrode system involving ISEs, ii) fluorometric readout of the sensor for activities of K^+^‐ion. b) i) Scheme illustrating the working of self‐powered bipolar electrode system, ii) sensor implementation with PB indicator toward glucose sensing. c–e) Schemes illustrating the implementation of cascaded bipolar electrode configuration with ISEs for fluorometric readout, colorimetric readout and memory‐based voltage readout. a) Reproduced with permission.^[^ ^97^ ^]^ Copyright 2018, Elsevier Ltd. b) Reproduced with permission.^[^ ^98^ ^]^ Copyright 2016, American Chemical Society. c) Reproduced with permission.^[^ ^102^ ^]^ Copyright 2020, Wiley‐VCH. d) Reproduced with permission.^[^ ^103^ ^]^ Copyright 2021, American Chemical Society. e) Reproduced with permission.^[^ ^22^ ^]^ Copyright 2021, American Chemical Society.

Jaworska et al. applied the strategy of self‐powered bipolar electrodes with ISEs to influence the emission signal of a fluorophore, which provided an optical readout for K^+^‐ion in solution (**Figure** **6**a).^[^ ^97^ ^]^ Using polypyrrole solid contact‐based K^+^‐ISE and zinc electrode, a bipolar system was created by establishing a short‐circuit between them in the same electrolyte. Due to a higher OCV of K^+^‐ISE than zinc electrode, a spontaneous process resulted in zinc oxidation and reduction of polypyrrole. Thereby, changes in K^+^‐ion activities resulted in an increase or decrease of zinc ion concentration. By forming a complex with 1‐(2‐pyridylazo)‐2‐naphthol (PAN) in the solution containing poly(n‐butyl acrylate) microspheres and fluorophore pyrene, the zinc ions influence the absorption spectrum of PAN contributing to an increase in fluorescence intensity of pyrene. Thereby, the obtained fluorescence intensity related linearly to the logarithm of the K^+^‐ion activity in solution.

Zhang et al. reported a self‐powered bipolar electrode array in a test paper‐like platform, where one end of BPE contained the element of interest, that is, in their case a catalyst or enzyme, and the other end featured a PB film to serve as a response indicator based on the spontaneous reaction induced color changes in the film (Figure 6b).^[^ ^98^ ^]^ The oxidation potentials of the involved reactions being sufficiently negative than the reduction potential (0.35 V) of PB film, they managed to screen different catalysts/enzymes and measure their activity based on the relative discoloring of the PB film. For instance, they performed glucose quantification by placing GDH enzyme as the anode component at one end of the BPE. When oxidized with glucose (−0.06 V), the system released electrons to facilitate reduction of PB film to PW changing the film's color from blue to white. The extent of the generated PW reflected the concentration of glucose.

Though spontaneous bipolar electrode systems offer straightforward and easy readout methodologies, they generate low junction potentials (usually a few millivolts) that limit their application to a narrow section of output indicators and electrochemical reactions.^[^ ^99^ ^]^ Additionally, the optical probes may experience contamination from the sample components and hence it is preferred to isolate the probe from the sample reservoirs.

### 5.4 Cascaded Bipolar Electrode Configurations

Cascading strategies with bipolar electrodes^[^ ^100^, ^101^ ^]^ offer promising solutions for generating higher voltages than that obtained with a single galvanic cell. In such a configuration, a self‐powered operation of one pair of electrodes (acting as driving electrodes) helps to increase the magnitude of induced voltage across a second electrode pair (in the sensing circuit) linked to detecting the analyte of interest by incorporating a spatially separated indicator and sensing probes.

Maksymiuk's group exploited this mechanism to obtain a fluorometric sensor readout of ascorbic acid or hexacyanoferrate (II) ions (HCF) using short‐circuited Pt and poly(3‐octylthiophene) (POT) coated electrodes in the sensing circuit.^[^ ^101^ ^]^ In the presence of separate short‐circuited driving electrodes (Zn and either K^+^‐ISE or Ag/AgCl), the oxidation of ascorbic acid or HCF at Pt electrode resulted in reduction of the polymer. This is due to the driving electrodes resulting in an induced voltage that was high enough to cause this redox process and generate a fluorescence signal corresponding to the analyte concentration. Based on a similar principle, the detection of Cl^−^ ions with ISEs has also been recently achieved by cascading bipolar electrodes (Figure 6c).^[^ ^102^ ^]^ Colorimetric self‐powered sensor system has been recently proposed in a cascaded bipolar electrode configuration with poly(vinyl‐chloride)‐based H^+^‐ISE using PB as an indicator (Figure 6d).^[^ ^103^ ^]^ The induced potential at the ISE due to the pH of a sample in one compartment was reflected by the color changes of PB film located in another compartment. By optimizing the system to generate color changes of PB film to sample induced voltages between 50 and 250 mV (versus Ag/AgCl), a wide pH measurement range (2–10.5) with a rapid response rate of 44 s was accomplished. The sensor's colorimetric response for different samples such as red wine, coke, coffee, baking soda, and antacid correlated well with a calibrated pH electrode.

In addition to the above‐mentioned optical readout systems, the higher induced voltages that result from using a cascaded bipolar electrode system can enable the operation of basic electronic components to derive self‐powered sensors with advanced functions. By interfacing a simple circuit comprising a capacitor and diode between the pair of bipolar electrodes in the sensing circuit, Sailapu et al. developed a memory‐based self‐powered potentiometric sensor (Figure 6e).^[^ ^22^ ^]^ The functioning of the sensor enabled the recording of a deviation in an analyte's concentration in a predefined interval by tracking the concentration profile exceeding a threshold value. The sensor harvests energy in the capacitor due to the pH‐induced voltage at the H^+^‐ISE. Without the diode, information about elapsed pH changes cannot be achieved as the capacitor voltage equals the induced OCV that only corresponds to the pH value at the given instant. Further, in the absence of driving electrodes, the induced potential between H^+^‐ISE and Ag/AgCl electrodes in the sensing circuit is not enough to operate the diode. So, with driving electrodes and the operation of a diode, the capacitor preserves the accumulated charge (acting like a memory element) despite the analyte returning to its original concentration following a perturbation over time. Thereby, information about any deviation in analyte concentration can be obtained by reading a single value of the capacitor voltage at the end of the pre‐defined interval.

The cascaded bipolar electrode configuration is an exciting prospect for sensing technologies because it operates without involving external power sources, allows for the spatial separation of electrodes, isolates the indicator from the sample, can deliver an optical response and suitable voltage for interfacing electronics. However, in some cases, an unsuitable driving voltage, the rapid exhaustion of the reactants, undesired species/reaction gradients across the cell, background signal, or excess spatial distribution may cause discrepancies and low amplitudes of output signal.^[^ ^99^ ^]^ Sensing strategies featuring a single sensor could minimize the overall design footprint of the device and reduce some of these constraints.

### 5.5 Intermittent Powering

Another interesting approach to operate high‐power consuming devices is to intermittently power them by periodically accumulating sufficient energy in a capacitor. Sode's group exploited this approach, where they used a single fuel cell to accumulate energy in a capacitor with the help of a charge pump integrated circuit (IC) for driving components with higher operating voltages and currents. The charge pump IC circuit operates with the input voltage produced through the enzymatic reactions in the fuel cell. It continuously accumulates charge across the capacitor to generate a voltage that is greater in magnitude than the input fuel cell's voltage. Once the voltage across the capacitor exceeds a certain “discharge start voltage”, the charge pump switches the role to allow the capacitor discharge by driving interfaced electronic circuit components. The capacitor supplies the power until its voltage reaches a defined “discharge stop voltage”, where the charge pump switches to its original task of accumulating the energy back in the capacitor. The frequency of this continuous charging and discharging depends on the fuel concentration that dictates the input power to the charge pump. As a result, this frequency can reveal the analyte concentration.

**Figure 7:**
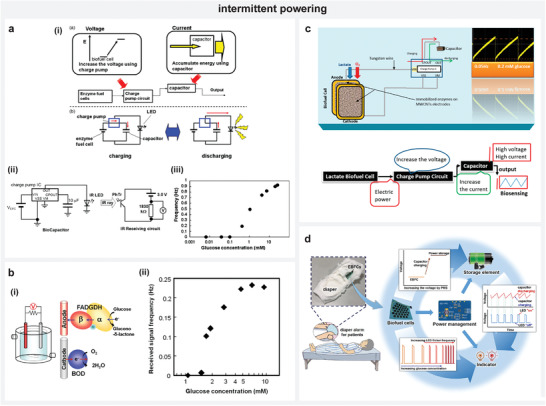
Intermittent powering. a) i) Scheme demonstrating the concept of intermittent powering using biofuel cell through cycles of charging and discharging the capacitor with IR LED as load, ii) self‐powered glucose sensing system with IR signal transmitting and receiving circuits, iii) sensor response in blinking frequency of the IR LED detected by IR phototransistor. b) i) Enzymatic cell used for the self‐powered detection of glucose, ii) sensor response relating the frequency of received signal (i.e., intermittently transmitted from the oscillator incorporating biofuel cell and charge pump) with glucose concentration. c) Lactate sensor working on the principle of intermittent powering. d) Self‐powered sensor based on the approach of intermittent powering implemented toward detection of sugar levels in urine. a) Reproduced with permission.^[^ ^104^ ^]^ Copyright 2008, Elsevier B.V. b) Reproduced with permission.^[^ ^105^ ^]^ Copyright 2011, Diabetes Technology Society. c) Reproduced under the terms of the Creative Commons CC‐BY license.^[^ ^110^ ^]^ Copyright 2017, The authors. Licensee MDPI, Basel, Switzerland. d) Reproduced with permission.^[^ ^111^ ^]^ Copyright 2021, Elsevier B.V.

With an enzymatic fuel cell that contained either glucose oxidase (GOx) or FAD‐dependent GDH (FADGDH) as an anodic catalyst, Sode's group observed that the frequency of capacitor charging or discharging cycles increased with glucose concentration, which enabled different readout modes by interfacing suitable output indicators. An infrared‐ light emitting diode (IR‐LED) and IR phototransistor‐based wireless glucose sensing system^[^ ^104^ ^]^ has been achieved with the capability of detecting glucose concentrations between 0.2 and 20 mm. The IR‐LED connected to the capacitor emitted a signal during the capacitor discharge phase, which was detected by the IR phototransistor. The frequency of this detected signal by the IR phototransistor corresponded to the glucose concentration (**Figure** **7**a). Similarly, they also developed a wireless BioRadioTransmitter biosensing system by interfacing Hartley oscillator^[^ ^105^ ^]^ and voltage‐controlled oscillators (VCO)^[^ ^106^ ^]^ that transmitted different frequency signals depending on the input power delivered by the fuel (Figure 7b). These signals were monitored by radio receivers to identify the glucose concentration. The group managed to run actuators,^[^ ^107^ ^]^ operate an ultra‐low power microcontroller^[^ ^108^ ^]^ and expanded their developments to develop a miniaturized glucose sensor^[^ ^109^ ^]^ based on this principle by incorporating a micro‐sized enzyme anode area of 0.1 mm^2^. Slaughter et al. developed a self‐powered lactate biosensor for real‐time monitoring of lactic acid by employing a similar principle (Figure 7c).^[^ ^110^ ^]^ The biofuel cell‐based sensor generated power by oxidizing lactic acid and reducing molecular oxygen with a D‐LDH modified anode and BOD‐modified cathode. The cell delivered an OCV of 395.3 mV, which was boosted to 1.2–1.8 V by the charge pump via the capacitor; the frequency of the voltage signal across this capacitor indicated the concentration of lactic acid. The sensor exhibited a sensitivity of 125.88 Hz mm ^−1^ cm^−2^ in a linear dynamic range of 1–100 mm lactic acid at physiological conditions. Recently, Zhang et al. exploited the concept of intermittent powering toward the detection of sugar levels using urine samples (Figure 7d).^[^ ^111^ ^]^ Their flexible enzymatic biofuel cell system on polyethylene terephthalate (PET)‐contained carbon nanotubes (CNT)/AuNPs hybrids with GOx as anode components and manganese dioxide (MnO_2_) as a cathode component. The fuel cell installed in the diaper‐generated power when contacted with urine to drive a LED through the power management system featuring a DC–DC boost converter. The flashing frequency of the LED revealed the sugar level in the urine.

While the power demands for operating components like LEDs, actuators and microcontrollers are often difficult to meet with in a single sensor, the strategy of intermittently powering them by charging a capacitor using charge‐pumps/DC–DC converters with SPES makes it possible to overcome this challenge. The only caveat to the approach of harvesting energy intermittently in a capacitor to enable operation of high‐power electronic devices is their non‐continuous operation.

### 5.6 Standalone Self‐Powered Devices with Naked‐Eye Readout

Electrochromic materials deliver an optical response based on the electrochemical oxidation state^[^ ^112^ ^]^ and can be exploited as response indicators to enable direct monitoring with the naked eye and thereby simplifying the readout process, especially in POC devices. They also share a similar configuration to fuel cells and batteries with a two electrode‐based system in a suitable electrolyte. Hence, they can form a part of sensing element alongside functioning as an indicator for the system. However, the selection of particular electrochromic materials involves considering various factors like its chemical nature, solubility, and optical switching potential.^[^ ^30^, ^113^ ^]^ For instance, an insoluble electrochromic material will prevent leaching and contamination of a sample ensuring stable sensor response. It is also extremely important to assess if the electrochromic material's color of interest is in oxidized or reduced form especially if the electrochemical material becomes one of the sensing electrode entities. In a self‐powered configuration, based on the counter electrode's reaction, the chosen electrochromic material should be in an electrochemical state that allows the opposite process to induce a spontaneous reaction.

**Figure 8:**
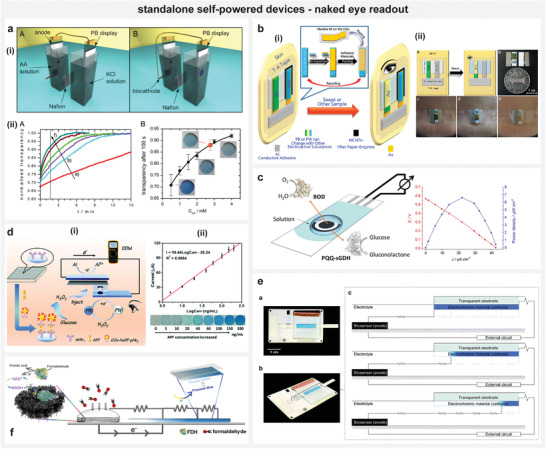
Optical based readout systems. a) i) Scheme illustrating a self‐powered ascorbic acid sensor with optical probe PB as one of the electrodes, ii) optical response of the sensor. b) i) Scheme representing a self‐powered glucose sensor, where the generated power drives an optical indicator. ii) Pictures showing the sensor operation. c) A self‐powered glucose sensor with optical readout on a flexible substrate. d) i) Scheme illustrates self‐powered POC immunosensor with optical readout, ii) optical response of the sensor toward detection of AFP. e,f) Self‐powered glucose and formaldehyde optical sensing based on relating length of color change on a strip to concentration of analyte. a) Reproduced with permission.^[^ ^113^ ^]^ Copyright 2013, Elsevier B.V. b) Reproduced with permission.^[^ ^114^ ^]^ Copyright 2018, American Chemical Society. c) Reproduced with permission.^[^ ^115^ ^]^ Copyright 2015, Elsevier B.V. d) Reproduced with permission.^[^ ^116^ ^]^ Copyright 2018, The Royal Society of Chemistry. e) Reproduced with permission.^[^ ^30^ ^]^ Copyright 2017, The Royal Society of Chemistry. f) Reproduced with permission.^[^ ^51^ ^]^ Copyright 2021, Elsevier B.V.

Zloczewska et al., demonstrated a self‐powered sensing system for determining ascorbic acid (AA, vitamin C) concentration in the sample by amalgamating an electrochromic display to reveal analyte‐related information based on color changes without involving any external instrumentation (**Figure** **8**a).^[^ ^113^ ^]^ The sensor, with carbon nanomaterial based non‐enzymatic anode to oxidize AA and BOD immobilized air‐breathing cathode, generated power in proportion to the AA concentration. However, to obtain an optical readout, only the anode of this biofuel cell was connected to the PB display located in a different compartment and separated by a Nafion membrane. PB is deep blue in oxidized state and becomes transparent in its reduced form. Typically, PB exhibits this color change at ≈0.4 V versus Ag|AgCl|KCl (3M) with a redox potential ≈0.2 V. So, when connected to the anode, PB was reduced to transparent form (PW) in the presence of AA, whose onset potential of oxidation is ≈0.05 V. The rate of this PB discoloration from blue to transparent form depended on the AA concentration in the sample. To regenerate the display to PB form, it can be connected to the cathode instead of anode whose onset potential for O_2_ reduction is higher (≈0.6 V) than PB. The authors successfully determined the AA concentration by analyzing the discoloration of PB‐display in a sample of orange juice when powered with the biofuel cell. In similar lines, Zhang et al., developed a flexible electrode based self‐powered POC fitness and athletic performance monitoring sensor capable of working with body fluids and delivering a visual readout using color transition of PB (Figure 8b).^[^ ^114^ ^]^ The sensor on a transparent adhesive tape incorporated Au/PB as the indicator on one of the flexible electrodes that coupled against the sensing electrode. They demonstrated the working of this sensing platform by placing it on skin toward detecting ionic strength, glucose, and lactic acid in sweat by using sensing electrodes as Al foil, Au/multi‐walled carbon nanotubes (MWCNTs)‐GDH, and Au/polymethylene blue‐MWCNTs‐LDH based electrodes, respectively. The color transition between PB and PW gave a simple visual readout of these parameters.

While the participation of the electrochromic material as one of the electrodes by making direct contact with sample makes the whole sensing system simple and compact, it can cause undesired sample contamination. One way to make the overall sensing system more robust is to drive the electrochromic material as a display indicator using an independently functioning electrochemical sensor. In an alternative approach, the power generated by the biofuel cell can be used to drive an electrochromic indicator that is not part of any analyte‐related reactions. Pinyou et al. developed a glucose sensing platform based on a biofuel cell, where the power generated by the cell reduced an electrochromic compound to deliver an optical readout (Figure 8c).^[^ ^115^ ^]^ Their miniaturized biofuel cell system was built on a screen‐printed electrode (SPE) with GDH‐ and BOD‐based electrodes for the catalytic oxidation of glucose and the reduction of molecular oxygen. With a small sample volume (≈60 µL) containing 10 mm glucose, the biofuel cell delivered an OCV of 567 mV with a maximum power density of 6.8 ± 0.6 µW cm^−2^. Further, the electrical power varied proportional to the glucose concentration (in the range of 0.1 to 1 mm). To provide an instrument‐free readout, the biofuel cell was connected to a second SPE, that one containing poly(3,4‐ethylenedioxythiophene) (PEDOT)‐modified methylene green (MG). MG shows a deep green/blue color that gradually turns transparent upon reduction. The discoloration of MG depended on glucose concentration, with a pronounced discoloration at higher glucose concentrations.

The color transition from PB to PW can easily be distinguished with the naked eye, which supports this method for being deployed in POC‐related immunoassays. Yu et al. employed a self‐powered electrochromic display with an Al/PB detection cell for POC‐related immunosensing of alpha‐fetoprotein (AFP, a biomarker for liver cancer) (Figure 8d).^[^ ^116^ ^]^ They initially performed a sandwich‐type immunoreaction with AFP in an anti‐AFP monoclonal antibody (mAb1)‐coated polystyrene microtiter plate using GOx and anti‐AFP polyclonal antibody‐labelled AuNPs (GOx‐AuNP‐pAb2) as the detection antibody. The binding of the AFP in the immunoreaction relates to the amount of GOx on the sensing platform. Further, on adding the glucose substrate, the AFP‐linked GOx catalyses the glucose decomposition, producing hydrogen peroxide, which serves as the input for the Al/PB detection cell. The hydrogen peroxide generated in this process changed the indicator status from PW to PB, giving a qualitative detection of the AFP with the naked eye. Though the above‐discussed optical detection techniques allow easy visualization of the sensor response, the majority provide only qualitative information with transient changes depending on the flowing current. These approaches still require involving some sort of image‐capturing or spectroscopy device to help deliver a semi‐quantitative or quantitative analysis.

To minimize cell resistance, conventional electrochromic displays usually employ a vertical architecture with the anode and cathode facing each other. Instead, by adopting a horizontal configuration, Pellitero et al. used the internal resistance of the cell advantageously and constructed a distance‐dependent meter, where the display first changes color near the vicinity of the anode following a least resistive path (Figure 8e).^[^ ^30^ ^]^ By using a PB‐based cathode and a GOx‐based anode, the authors developed a cell that produced power proportional to analyte concentration with a maximum power density of ≈13 µW cm^−2^. The authors further showed that the glucose concentration can be correlated to the length (or distance) of the color change at the PB‐based cathode. This design of electrochromic display enables a semiquantitative measurement by plain sight, akin to a metering bar, due to the geometry of the cell. Very recently, based on a similar approach, Sun et al. developed a self‐powered sensor for detecting gaseous formaldehyde, an ubiquitous air pollutant in the human living environment, using PB as an indicator, thus enabling a naked eye readout (Figure 8f).^[^ ^51^ ^]^ The sensor was patterned on ITO glass substrates with formaldehyde dehydrogenase (FDH)/poly(methylene green) (PMG)/buckypaper (BP) bioanode and a PB cathode. By covering the sensor with poly(vinyl alcohol) (PVA) as a gel electrolyte, a large lateral resistance was created to enable the progressive discoloration of PB when exposed to formaldehyde.

### 5.7 Digital and Wireless Devices

Although optical based readout systems, particularly those which can be read out by the naked eye offer exciting prospects, they deliver only minimal information such as a “yes/no” or semi‐quantitative analysis which is often subjective to human error. Standalone devices that can provide a digital readout prove useful to acquire quantitative, or reliable and accurate semi‐quantitative information. Most of the optical self‐powered devices described above can be coupled with smartphones or handheld portable readers to capture and further process the images or optical signals with suitable software and hardware implementation for acquiring semi‐quantitative or quantitative information. Alternatively, standalone devices that can accomplish this task on a freely‐behaving sensing platform offer a convenient solution relying less on external readers. However, at the same time, this process could yield bulky and costly devices featuring many components. This could lead to great inconvenience in the case of wearable sensors and could impact the environment negatively by accumulating e‐waste, especially in the case of disposed POC‐related devices.

**Figure 9:**
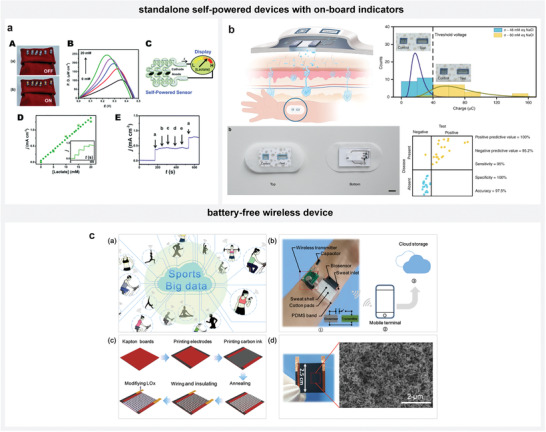
Digital and wireless devices. a) Stretchable textile‐based autonomous sensor (STAS) that can detect the lactate level in sweat and scavenge energy from sample to indicate the response on an ammeter display. b) Self‐powered wearable skin patch to measure conductivity in sweat and to display the result on an electrochromic display by deriving the required power from the sample itself. c) Self‐powered wearable sweat‐lactate analyzer to build sports big data through a wireless transmission system that is run by the power generated with the analyzed sweat sample. a) Reproduced with permission.^[^ ^117^ ^]^ Copyright 2016, The Royal Society of Chemistry. b) Reproduced under the terms of the Creative Commons CC‐BY license.^[^ ^28^ ^]^ Copyright 2019, The Author(s). Published by Springer Nature. c) Reproduced with permission.^[^ ^53^ ^]^ Copyright 2019, Elsevier Ltd.

One way to avoid the device design drawbacks listed above is to implement a freely‐behaving sensing platform. Jeerapan et al. demonstrated a stretchable textile‐based autonomous sensor (STAS) that can “sense” lactate level in sweat and further “scavenge” energy from the sample to “display” information about the analyte in an analog ammeter display (**Figure** **9**a).^[^ ^117^ ^]^ The self‐powered sensor employed for this task is a biofuel cell hosting a bioanode functionalized with LOx to promote the oxidation of lactose in sweat, and a cathode based on silver(I) oxide/silver (Ag_2_O/Ag) redox couple to accept the generated electrons. Incorporating screen‐printing technologies, the authors showed that these sensors on textiles can withstand stretching, indentation and twisting by including nanomaterial‐based engineered inks and serpentine designs. The sensor generated an OCV of 0.46 V and maximum power density of 250 µW cm^−2^ with 20 mm lactate. By connecting the sensor directly to an analog ammeter display, the short‐circuited current obtained with the stretchable biofuel cell was quantified. Thereby, the system demonstrated a concept of “scavenge‐sense‐display” by extracting the energy from sweat to sense lactate and display the quantified information on an analog ammeter. The method detected lactate up to 20 mm with a detection limit of 0.3 mm and sensitivity of 66.5 ± 6.8 µA cm^−2^ mm ^−1^. Analog signal related displays can sometimes be tedious to read and digital displays are widely preferred for enabling easy interpretation of the POC test result. Ortega et al. developed a wearable self‐powered skin patch that is capable of delivering a digital result on an on‐board electrochromic display using a simple approach and by incorporating only a few electronic components (Figure 9b).^[^ ^28^ ^]^ The patch contained a paper battery constructed of magnesium (anode), silver chloride (cathode) placed on top of a pressure sensitive adhesive layer and connected through a glass fiber‐based paper. The paper battery delivered power ranging between 0.2 and 2 mW in proportion to the conductivity of the liquid sample. The paper battery absorbed sweat and used it as the electrolyte to produce the power corresponding to the conductivity of sweat. This behavior made it feasible to derive a conductivity response by using the battery itself as a sensor in a direct current mode instead of employing the AC current generally required for conductivity measurements. Thereby, the power generated from the sweat‐activated battery drove a very simple electronic circuit that discerned a healthy and non‐healthy condition in an unambiguous manner by indicating the result in an electrochromic display. The patch delivered results with 95% sensitivity and 100% specificity with artificial eccrine perspiration samples.

Dedicated power sources in POC diagnostic devices, like batteries, not only add to the cost and weight of the device but their unregulated disposal can also cause an environmental issue. Making POC devices self‐powered by incorporating only the minimum of components required to deliver a digital response offers a portable, low‐cost, and environmentally friendly solution. Merino‐Jimenez et al. recently demonstrated a standalone self‐powered biosensing device with a digital readout for screening gestational diabetes mellitus by involving no external power sources.^[^ ^12^ ^]^ The device contained a paper‐based enzymatic fuel cell with an FAD‐GDH‐based anode and an Ag*_x_* O‐based cathode to function as both power source and sensing component. The detection strategy involves transferring the generated charge—via the oxidation of glucose in the sample—to an electronic capacitor. The fuel cell generated an OCV of 0.85 V that dropped initially to 0 V when interfaced to a capacitor, to eventually build up a voltage across capacitor over time depending on the analyte concentration. By correlating the built‐up capacitor voltage in this process at a specific time, the analyte concentration was revealed. The device further employed an electro‐fluidic switch to connect a minimalistic electronic circuit to the fuel cell for displaying a semi‐quantitative result on an electrochromic display by analyzing the built‐up capacitor voltage. Overall, the device uses only 3.5 µL of sample to power the entire diagnostic device and to indicate the healthy, pre‐diabetic, or diabetic condition.

Instead of an on‐board display‐based response indicator, incorporating battery‐free wireless readout technology could provide a smart and easy approach, especially in wearable devices. Particularly, RF and NFID‐based technologies offer promising applications for long‐distance reading, continuous data acquisition, and the convenient installation of readers at desired appropriate locations. Guan et al. demonstrated the feasibility to build sports big data by integrating a wireless transmitter that is entirely run by the generated power in the self‐powered wearable sweat‐lactate analyzer (Figure 9c).^[^ ^53^ ^]^ The sensor fabricated from LOx‐modified porous carbon film worked based on a sweat–evaporation–biosensing coupling effect. As the sweat contacted the porous carbon film on the device, an output voltage was generated based on the ion dependence of NaCl and the enzymatic reaction. When attached to the athlete's skin, the self‐powered sensor generated a voltage that increased with an increase in lactate concentration. The energy generated by this process can be stored in a capacitor to drive a wireless transmitter for transmitting the response to external platforms like wireless receivers or mobile phones. Often, knowing about more than one physiological parameter helps to obtain a broad understanding of the physiological status of an individual. Recently, Bandodkar et al. developed a non‐invasive, wearable, battery‐free wireless electronic sensing platform for simultaneously monitoring several physiologically important parameters like glucose, lactate, pH, chloride, and sweat rates that can aid in providing semi‐quantitative information about an individual's health.^[^ ^29^ ^]^ Besides colorimetric assaying for detecting chloride and pH, the sensor featured enzymatic biofuel‐based glucose sensor and lactate sensor by incorporating separate anodes modified with LOx and GOx enzymes, respectively. When assembled against a current collecting cathode, the target analytes spontaneously generated an electrical current relating to their concentration in the sweat sample. By connecting a suitable resistive load across these self‐powered sensors, a voltage readout was obtained corresponding to this current. The authors further demonstrated the possibility of real‐time data acquisition from these sensors by initiating the readout process wirelessly through magnetically coupling the NFC electronic system embedded in the sensing platform through a suitable reader.

## 6 Challenges, Prospects, and Opportunities

**Figure 10:**
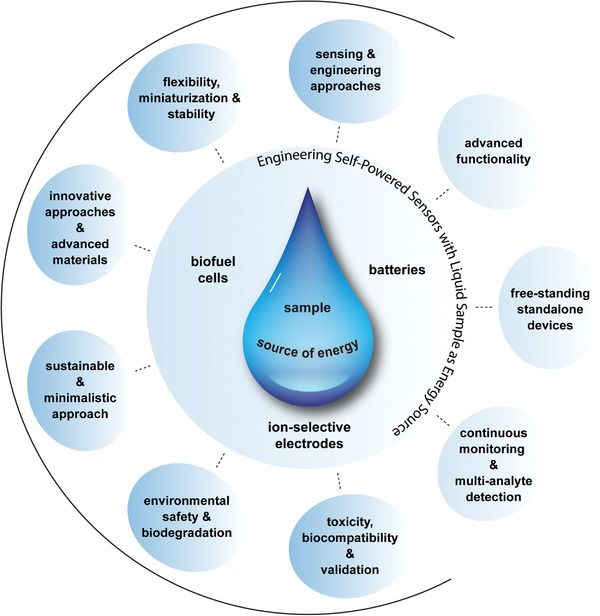
Challenges, prospects, and opportunities with SPESs.

From the initial simple realization of self‐powered sensors with monitoring OCV, current or power through a resistive load as output, the field of SPES has taken major strides toward architecting free‐standing standalone devices. While this marks a significant development in the field of sensing with SPES, its advancement can be propelled further by addressing a few more challenges to appreciate the enormous potential and opportunities that these systems can offer (**Figure** **10**).

### 6.1 Innovative Approaches and Advanced Materials

When operating in challenging environments that present dynamically changing parameters, the sensor may fail to work or experience a reduction in its overall performance. For instance, enzymatic biofuel cell‐based sensors remain one of the most popular areas in SPES research due to the excellent specificity, selectivity, and sustainability offered by biocompatible enzymes. Particularly, they help to realize sensors for operating in wearables and implantable devices. However, most of these enzymes are known to experience natural degradation, hampering their stability. Thereby, efforts must be laid in future for improving their resistance to degradation or pursuing novel strategies involving conductive enzyme‐like biomolecules.^[^ ^118^ ^]^ Further, improvements in DET mechanisms by engineering an enzyme‐electrode surface with conductive materials would help to enhance the electrochemical performance of enzyme‐based sensors. Moreover, instead of involving a single enzyme for oxidation of complex molecules, it is beneficial to improve energy conversion through cascaded approaches by combining them with organic and inorganic catalysts.^[^ ^32^, ^119^ ^]^ Considerable issues with other self‐powered systems that seek improvements are related to solving the solubility problems with electrochromic materials,^[^ ^120^ ^]^ membrane fouling and degradation with ISEs. The sensors must as well be prepared to function efficiently in challenging conditions such as low oxygen, limited fuel, fluctuating temperatures and pH, by employing advanced materials or innovative approaches to reduce any direct dependency (e.g., reactions devoid of oxygen) of sensor on these parameters. Thereby, constant improvements have to be sought in electrode design, novel materials, efficient and faster electron transfer mechanisms, and higher material loading efficiencies to propel the growth of SPES.

### 6.2 Continuous Monitoring

Since self‐powered devices can derive a source of energy from components in body fluids, they hold immense potential for developing continuous healthcare monitoring devices, artificial organs, and smart devices for supporting body functions. Due to the availability of various organic and inorganic resources, metabolites and ions, the biological medium is a promising environment for deploying most of these SPES. While it is an exciting opportunity to envision such devices, it requires overcoming few challenges. Particularly, to accomplish a prolonged operation or continuous monitoring, these devices must be compatible in the first place and should not be rejected by the body. Besides, it is necessary to ensure that the by‐products from sensor reactions do not induce any undesired side‐effects. While the bio‐environment is resourceful, it as well hosts many other entities in the complex environment which can cause the sensor operation to cease or perform at reduced efficiency. Other related factors, for example, pH, proteases, in operating medium as well interfere with the sensor functioning. Hence, it is essential to consider all these elements while designing the SPES as choice of materials and relevant sensing approaches would impact its efficient functioning in challenging conditions. It is also essential to secure the device and sensor components from plaguing while operating in bio‐related environments or at a semi‐bio interface. Amongst others, continuous efforts must also be made to improve the response rate, sensitivity, and quality of output signal from SPES.

Organic mediators, exogenous coenzymes, metals, various polymers, and redox indicators often feature in the design of either the electrode or the electrochemical system. While they aid in improving the overall functionality of the device, the wide application of SPES would be limited if it is deemed hazardous or toxic. So, a systemic study of their compatibility and toxicity on humans (in vitro and in vivo) must be carried out to assess their overall applicability, especially to wearable technologies and implantable devices.^[^ ^32^, ^34^ ^]^ Also, in case of disposable devices or those intended for single‐use applications, a proper assessment is necessary to ensure environmental safety and biodegradation. Specifically, for devices that are deployed in water bodies such as rivers, a prior study of their effect on aquatic life and contribution to water pollution must be made.

### 6.3 Miniaturized and Flexible Systems

Traditional systems with large solid electrodes are well‐established in electrochemical based sensors for performing a variety of analytical techniques with ease while ensuring good reproducibility and repeatability. But these systems require miniaturization to suit POC tests and portable devices. This process may well affect the overall sensor stability and performance, particularly when operating with low volumes of liquid sample in POC‐related sensing devices or wearables. Further, challenges related to achieving a stable miniaturized reference, fast responding sensing electrodes, strategies for efficient storing of reagents must be addressed. Printing technologies and rapid prototyping approaches provide simple tools for accomplishing reliable miniaturized electrodes allowing for the further processing of electrode surface, immobilization with suitable materials, and interfacing with commercial electronic components. These miniaturized systems have to be carefully tailored to produce sufficient electrical or optical signals desired for the application. Moreover, large‐solid electrodes and rigid platforms can also lead to inconvenience for users and may cause critical issues in implantable devices by resulting in the chocking or clogging of blood vessels. Alternatively, implementation of flexible and biodegradable substrates that can support the sensing components is desirable for such situations. However, it then becomes necessary to ensure that the bending/twisting of the substrate do not incur any damage to the sensor itself.

### 6.4 Advanced Sensors, Standalone Devices with Sustainable and Minimalistic Approach

Besides investigating suitable electrodes and materials to improve the overall sensor performance, future research must tackle the challenges of expanding the breadth of these sensors to real‐life practical applications. This particularly requires smart sensing strategies and engineering approaches to deal with the low power available from these sensors without introducing complicated approaches and keeping the entire sensing device operation simple. Not a lot of research works have dealt with these challenges and future research in relation to device development require investigations on proper system integration, minimal electronics compatible to printed technologies, and data and storage management. Importantly, the devices are preferred to abide the REASSURED criteria (Real‐time connectivity, Ease of specimen collection, Affordable, Sensitive, Specific, User‐friendly, Rapid and robust, Equipment free or simple Environmentally friendly, Deliverable to end‐users).^[^ ^121^ ^]^ While a considerable progress has been achieved in sensing different analytes, it is time to pursue to an advanced form of sensors that can give some sort of additional functionality, for example, tracking events (memory‐based) without continuous monitoring or devices capable of disease diagnosis, treatment and prognosis. Although several analytes have been detected with SPES, only a few works have attempted detection of multiple analytes on a single platform. For instance, simultaneous information about different biomarkers can help to achieve a better diagnosis, treatment, and prognosis.

## 7 Conclusion

Overall, the current review presented various sensing and engineering approaches using SPES, their related benefits and drawbacks. **Table** **1** summarizes these approaches, indicating the related sensing systems in each category. Further, we discussed these approaches in the context of three different electrochemical systems that use the analyzed liquid sample as the sole energy source: biofuel cell, battery, and ISE‐based systems. These two‐electrode‐systems without featuring an external power source to trigger sensing‐related reactions, allow for easy miniaturization, and can operate with a wide range of liquid samples. Importantly, they hold the potential to deliver a sustainable technology with a scope for considerable improvement in the field of self‐powered sensing and related areas.

**Table 1:** Various sensing and engineering approaches using SPES

Categories	Approaches	SPES					
		Sensing component	Type of sensor	Prominent cell component		Output type	Ref.
				Anode (+ve)	Cathode (−ve)		
Voltage/current/power directly relates to analyte concentration	Voltage as output	Cancer cells	Enzymatic	PQQ‐GDH	BOD	Voltage	[76]
		Conductivity	Battery	Mg	Ag/AgCl	Voltage	[46]
	Current as output	Ethanol	Enzymatic	NAD^+^‐ dependent ADH	AOx + HRP	Current	[60]
		Lactate	Enzymatic	LOx	BOD	Current	[59]
	Power as output	Glucose	Enzymatic	GOx	Pt	Power	[47]
Generated energy enables measuring of second analyte in the same sample		Glucose	Battery	Al	C	Colorimetric	[48]
		Glucose	Enzymatic	GDH	BOx	Current	[85]
		Lactate	Enzymatic	GOx	PB	Current	[86]
		HIV‐1 p24	Enzymatic	FAD‐GDH	Silver oxide	Voltage	[49]
Engineering strategies for self‐powered sensing	Harvesting energy in a capacitor	Adenosine	Enzymatic	GOx	[Fe(CN)_6_]^3−^	Current	[89]
		DNA	Enzymatic	GOx, HRP	Pt nanoparticles	Current	[81]
		PSA	Enzymatic	Cu_2_O‐TNTs	BOD	Current	[90]
		K^+^	ISE	K^+^‐ISE	K^+^‐ISE (ref)	Voltage	[21]
	Bipolar electrode configurations	K^+^	ISE	Polypyrrole solid‐contact K^+^‐ISE	Zn	Fluorometric	[97]
		Glucose	Enzymatic	GDH	PB	Colorimetric	[98]
	Cascaded bipolar electrode configurations	Cl^−^	ISE	Driving electrodes: Zn, K^+^‐ISE Sensing electrodes: POT, Cl^−^‐ISE		Fluorometric	[102]
		pH	ISE	Driving electrodes: Zn, Ag/AgCl Sensing electrodes: H^+^‐ISE, Ag/AgCl		Voltage	[22]
	Intermittent powering	Glucose	Enzymatic	FAD‐GDH	BOD	Voltage (frequency)	[105]
		Lactate	Enzymatic	D‐LDH	BOD	Voltage (frequency)	[110]
	Optical readout	Ionic strength	Battery	Al	PB	Colorimetric	[114]
		Glucose	Enzymatic	GDH	BOD	Colorimetric	[115]
		AFP	Enzymatic	GOx	Al/PB	Colorimetric	[116]
		Formaldehyde	Enzymatic	FDH/PMG/BP	PB	Colorimetric	[51]
	Digital and wireless devices	Lactate	Enzymatic	LOx	Ag_2_O/Ag	Current (analog ammeter)	[117]
		Conductivity	Battery	Mg	Ag/AgCl	Voltage (electrochromic display)	[28]
		Glucose/lactate	Enzymatic	GOx/LOx	Platinum black	Voltage (NFC)	[29]

The field of SPES has advanced in recent years, tackling various challenges and making continuous progress in realizing systems with good electrode stability, efficient electron transfer, chemical inertness, wide OCV, large current and power densities,good mechanical flexibility and robustness. Despite SPES offering low electrical power, significant efforts to perceive compatible sensing devices using interesting readout approaches for application in POC devices with low sample volumes, wearable platforms, and implantable devices is encouraging for further advancements in the field. SPES are an exciting prospect with a very broad range of applications related to healthcare, agriculture, defense, the environment, and other fields. It is crucial to engineer these sensors for enabling concurrent detection of multiple analytes, developing advanced sensor‐related functions, and implementing hybrid systems. Most importantly, SPES should be integrated, enabling free‐standing standalone device operation in a portable form, while harnessing all the required power from the analyzed liquid sample itself.

## Conflict of Interest

The authors declare no conflict of interest.
